# Effects of seniority, gender and geography on the bibliometric output and collaboration networks of European Research Council (ERC) grant recipients

**DOI:** 10.1371/journal.pone.0212286

**Published:** 2019-02-14

**Authors:** David G. Pina, Lana Barać, Ivan Buljan, Francisco Grimaldo, Ana Marušić

**Affiliations:** 1 Research Executive Agency, European Commission, Brussels, Belgium; 2 Research Office, University of Split School of Medicine, Split, Croatia; 3 Department of Research in Biomedicine and Health, University of Split School of Medicine, Split, Croatia; 4 Department of Computer Science, University of Valencia, Valencia, Spain; Max Planck Society, GERMANY

## Abstract

Assessing the success and performance of researchers is a difficult task, as their grant output is influenced by a series of factors, including seniority, gender and geographical location of their host institution. In order to assess the effects of these factors, we analysed the publication and citation outputs, using Scopus and Web of Science, and the collaboration networks of European Research Council (ERC) starting (junior) and advanced (senior) grantees. For this study, we used a cohort of 355 grantees from the Life Sciences domain of years 2007–09. While senior grantees had overall greater publication output, junior grantees had a significantly greater pre-post grant award increase in their overall number of publications and in those on which they had last authorship. The collaboration networks size and the number of sub-communities increased for all grantees, although more pronounced for juniors, as they departed from smaller and more compact pre-award co-authorship networks. Both junior and senior grantees increased the size of the community within which they were collaborating in the post-award period. Pre-post grant award performance of grantees was not related to gender, although male junior grantees had more publications than female grantees before and after the grant award. Junior grantees located in lower research-performing countries published less and had less diverse collaboration networks than their peers located in higher research-performing countries. Our study suggests that research environment has greater influence on post-grant award publications than gender especially for junior grantees. Also, collaboration networks may be a useful complement to publication and citation outputs for assessing post-grant research performance, especially for grantees who already have a high publication output and who get highly competitive grants such as those from ERC.

## Introduction

Peer review remains the core paradigm in assessing different research activities despite contradictory evidence on whether it is the best way of selecting grant proposals and judging articles' suitability for publication [1]. In addition, there are a few studies about the effects of receiving a grant on a researcher's performance [2–3]. Nevertheless, research into peer review of funding agencies is key as it can bring about policy changes or process improvements, possibly resulting in better use of resources [4].

Even though peer review should be objective, many biases were reported to affect it [1,5]. Among them, gender has been identified as a part of the general disparity in productivity between male and female researchers at all stages of their professional careers [6–10]. In several research fields, female researchers obtain lower funding [11–12] and, even when receiving similar grant funding, female faculty may lag behind their male colleagues in terms of publications and citations [13]. Longitudinal studies have shown that gender bias exists over professional research careers, even after controlling for other factors, such as research field and performance differences [6]. Another important bias–geographical location of the researcher host institution–has also been described as influencing the peer review process in scientific journals [14] and in research grants, even within a single country [15].

It is not clear whether standard bibliometric indices, such as publications and citations, are adequate measures of grant success and performance [16–17]. This uncertainty stems not only from the evidence that publications and citations outputs are diverse and vary between research disciplines [18], but also because there is conflicting data on their value as measures of grant success [19–20]. While some studies found a correlation between higher grant proposals review scores and grant output measured as citations and patents [3, 21–22], others have failed to directly confirm the importance of these outputs as a validation measure of the grant peer review process [2,23]. The assessment of grant success may be even more challenging for highly competitive grants, where the choice of the best among the best is very difficult. A possible alternative for assessing grant success could be the analysis of co-authorship networks, because it puts the individual research performance into a wider social context. Co-authorship networks establish collaboration patterns among scientists by using data drawn from their publication record [24–25].

The aim of our study was to assess seniority, gender and country differences in publication performance for a sample of grantees from the European Research Council (ERC), the EU flagship research funding agency. ERC's peer review process has been well described [26–28], with a recent study showing that it attracts high profile researchers [29]. However, that study did not establish a strong evidence for a major quantitative or qualitative impact on the publication output of grantees. Therefore, we analysed, for a cohort of junior and senior life sciences' ERC grantees from the years 2007–2009, both the publications/citations outputs and collaboration networks in the 5-year period before and after the award of their grants. We were particularly interested in the change of publication performance in relation to the grant award and whether the type of award (seniority), gender, and geographical location (higher vs. lower research performing countries) are associated with these differences.

## Methods

**Fig 1:**
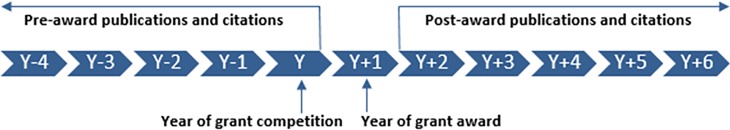
Schematic illustration of the time frames used for the extraction of data on publications and citations. Y indicates the year of the grant competition, and Y+1 the year of the grant award. Pre-award publications were extracted for 5 years, including the year of the grant competition. The post-award period was considered as the 5 years after Y+1, where the publications related to the grant could be expected. The same periods were used to collect the citations of the extracted publications.

For the purpose of this study, we used the data publicly available at the ERC website (https://erc.europa.eu) for a cohort of 355 grantees from the years 2007–2009, in order to select those who would have completed their grants by the end of 2015. We focused our study only on the life sciences grantees, because research output in this field is mostly observed through the publications in peer-reviewed journals, which are well covered by the Scopus and Web of Science Core Collection (WoS) databases [18]. This cohort was composed of Starting Grants (StG), designed to support *junior* researchers at the stage at which they are starting or consolidating their own independent research team, and Advanced Grants (AdG), reserved for leading *senior* investigators, having a track-record of significant research achievements in the last 10 years. Both grant types have a 5-year average duration (in our sample, 61% of grants lasted for 5 years, and 98% lasted for 4 to 6 years), and are awarded using a review process sharing common evaluation standards. For each grantee, a publication search was performed for the 5-year period before and after the year of the award of the grant (**Fig 1**). Individual grantee names from the list of the awarded ERC grants in the Life Sciences domain were used for “Author search” in the Scopus and WoS databases to identify publications for each individual (articles and reviews in English only). The gender of the grantees was judged by two authors (DGP and LB); in cases of ambiguity, a web search was performed to find possible identifying information, and a third author was consulted (AM) for final agreement. Geographical grouping was based on the host organisation location (not the grantee's nationality), and the split between the higher research-performing and lower research-performing countries was made according to the composite indicator for research excellence defined by the EU [30]. Countries considered as high-research performers were those with an indicator above the global EU value (Austria, Belgium, Denmark, Finland, France, Germany, Israel, Netherlands, Norway, Sweden, Switzerland, UK), whereas the ones considered low-research performers were those below that value (Bulgaria, Croatia, Cyprus, Czech Republic, Estonia, Greece, Hungary, Ireland, Italy, Latvia, Lithuania, Luxembourg, Malta, Poland, Portugal, Romania, Slovakia, Slovenia, Spain, Iceland, Turkey).

The data were collected for StG and AdG recipients separately, and gender and country differences analysed separately for each group. Grouping of the two types of grants for the output analysis was not attempted because of large differences in publication and citations outputs.

For the analysis of the scientific collaborations established by individual grantees in the periods before and after the award of the grant, we constructed collaboration, i.e. co-authorship, networks [24–25, 31], derived from the publications retrieved in this study from Scopus for each period. We used the set of documents extracted from Scopus only since the results of searching this database are more complete and they allow us to build more robust networks. We had no access to the content of the proposals and could not establish whether the co-authors identified were also named as collaborators in the grant proposals. Nodes in these networks represent different researchers and edges connect two of them if they have co-authored a paper. The basic network indicators were:

1. *Number of different co-authors*. This indicator corresponds to the number of nodes in the network and, therefore, to the number of different co-authors found in the total number of publications for the period under consideration. It then measures the size of the research community the grantee is collaborating with before and after the grant.

2. *Number of co-authorships*. This indicator corresponds to the number of edges in the network or, similarly, to the sum of all different two-by-two relationships between researchers that can be generated from the list of co-authors of each paper published in the period under examination. This metric represents the global amount of collaboration generated by the papers published by the grantee.

We also calculated the following indicators:

3. *Network density*. This is the ratio between the number of edges in the network and the total number of edges if the network was completely connected. It measures how compact the co-authorship network is. The less compact it is, the more diverse the publication pattern will be.

4. *Number of sub-communities*. This is the number of densely connected clusters in the co-authorship network. We quantified how structured the community is by calculating the leading non-negative eigenvector of the community matrix [25].

5. *Network modularity*. This indicator measures how good the previous division into clusters is, or how separated are the different members of the sub-communities from each other. Modularity is calculated as the fraction of within-community edges minus the expected fraction if edges were distributed at random. The value of the modularity lies in the range −1 to 2.1 and, in practice, a value above 0.3 is a good indicator of significant community structure in a network [32].

6. *Grantee eigencentrality*. This is a measure of the influence of the grantee in the collaboration network. The normalized eigenvector centrality defines a ranking over the set of researchers in the network by assigning relative scores to all nodes based on the concept that connections to high-scoring nodes contribute more to the score of the node in question than equal connections to low-scoring nodes [33]. This means that a researcher is important if he or she is linked to other important researchers. In this paper we observe the evolution of the centrality that is assigned to grantees before and after the grant.

*7*. *Network centralization*. Centralization is a method for creating a network level centralization measure from the centrality scores of the researchers. It measures how much variation there is in the eigencentrality scores among the nodes [33]. Thus, in a similar way to the Gini index [34], it quantifies how equal the researchers are in the collaboration network defined by the papers co-authored with the grantee.

R programming language version 3.4.4 with iGraph library version 1.1.2 was used to perform the co-authorships networks analysis.

Statistical analysis was performed using MedCalc version 17.1 (MedCalc Software, Ostend, Belgium) and R programming language. The results were expressed as medians with 95% confidence intervals. Comparisons of indicators for individual groups at pre- or post-award time periods were based on the interpretation of 95% confidence intervals, where non-overlapping confidence intervals indicated that the measured values belonged to different populations and were therefore statistically significant. Comparison of differences between the pre- and post-award indicators for different groups was performed using Mann-Whitney U test. In a complementary analysis, we also used Bayesian t test for independent samples. Bayes factors were calculated using JASP 0.8.3.1 (JASP Team, 2017) assuming a default prior distribution [35]. Bayes Factors (BF10) with values which remained above 3 after sequential analysis and robustness check were considered to indicate substantial evidence for the alternative hypothesis [36]. In cases of discrepancies between frequentist and Bayesian statistics, we used the Bayesian approach to interpret the significance of the results, due to the inequalities in sample size and in order to quantify the size of the evidence for the tested hypothesis [35]. Moreover, due to the use of uninformative prior, we presumed that, if there was a real difference between the groups, the results of the frequentist and Bayesian analysis would not differ.

## Results

Overall, most of the grantees were male (n = 291, 82%), both for StG (n = 144, 78%) and AdG groups (n = 147, 86%) (P = 0.931). Also, most of the grants were awarded to researchers from higher research-performing countries (n = 299, 84%). The median funding was 1.2 (95% CI 1.2–1.4) and 2.2 (95% CI 2.1–2.3) million € for StG and AdG, respectively. There was no difference between junior and senior grantees in the mean publication cost from the grant (total grant funding divided by the number of publications after grant award): €63,000 (95% CI €52,800-€70,300) vs €56,900 (95% CI €50,000-€62,500) (P = 0.080).

### Effect of seniority

Most of the publications, for both types of grantees, fell in the first quartile of Scopus-indexed journals (median 93%, 95% CI 92–94% for StG; median 92%, 95% CI 81–94% for AdG). In absolute terms, senior grantees had more publications than junior grantees, both in the pre- and post-award periods (**Table 1**). However, in relative terms, junior grantees had a significantly greater increase in the number of publications from the pre*-* to the post*-*award period (**Table 1**). Before the grant award, senior grantees published more manuscripts as last (senior) author than junior grantees, but this difference disappeared after the grant award, as junior grantees had a significant increase in the proportion of publications with last authorships (**Table 1**). For all grantees, the increase in the number of publications in the post-award period was not accompanied by major changes in the median number of citations per publication (**Table 1**).

**Table 1:** Publications and citations (median, 95% confidence interval) by junior (StG) and senior (AdG) ERC grantees in the pre- and post-award period^a^.

Database^b^			StG (n = 184)			AdG (n = 171)			
		Pre-award	Post-award	Difference	Pre-award	Post-award	Difference	P^c^	BF_10_^d^
**Scopus**	**No. of publications**	11.0 (10.0, 13.0)	20.0 (17.0, 22.0)	7.0 (6.0, 8.2)	33.0 (29.0, 38.0)	37.0 (32.0, 43.0)	3.0 (0.0, 4.4)	<0.001	0.2
	**Citation per publication**	19.3 (17.1, 21.7)	15.6 (13.7, 17.9)	-2.8 (-1.0, -4.7)	20.8 (19.2, 22.4)	18.7 (17.1, 20.5)	-1.11 (-0.1, -2.7)	0.032	1.6
	**% last authorships**	18.8 (13.5, 25.0)	52.1 (48.7, 56.6)	21.3 (16.4, 21.3)	50.0 (46.9, 52.9)	48.3 (43.8, 51.1)	-4.1 (-6.3, -0.4)	<0.001	**3.7x10**^**24**^
	**Authors per publication**	5.0 (5.0, 6.0)	6.0 (5.5, 6.6)	1.0 (0.5, 1.0)	6.0 (5.0, 6.0)	6.5 (6.0, 7.0)	1.0 (1.0, 1.0)	0.528	0.2
**Web of Science**	**No. of publications**	11.0 (10.0, 12.8)	19.0 (16.0, 20.8)	6.0 (5.0, 8.8)	31.0 (27.0, 36.0)	37.0 (31.2, 41.0)	2.0 (0.0, 5.0)	0.004	0.1
	**Citation per publication**	18.1 (16.4, 20.9)	15.3 (13.0, 17.7)	-3.1 (-4.8, -1.7)	19.8 (19.1, 21.7)	17.5 (16.6, 19.8)	-1.6 (-3.5, -0.4)	0.069	1.1
	**% last authorships**	19.1 (15.4, 25.0)	51.6 (47.5, 54.9)	24.4 (16.8, 30.0)	48.4 (46.2, 52.0)	49.5 (43.6, 51.0)	-3.9 (- 6.7, -1.7)	<0.001	**2.6x10**^**21**^
	**Authors per publication**	5.0 (5.0, 6.0)	6.0 (6.0, 7.0)	1.0 (0.5, 1.0)	6.0 (5.5, 6.0)	6.5 (6.0, 7.0)	1.0 (0.8, 1.0)	0.669	0.2

^a^See Methods section and **Fig 1** for explanations about timeframes for data collection.

^b^Scopus and Web of Science Core Collection bibliographic and citation databases.

^c^Comparison of median post- vs. pre-grant differences between StG and AdG, Mann-Whitney U test for independent samples.

^d^Comparison of median post- vs. pre-grant differences between StG and AdG, Bayesian t test for independent samples (significant differences in bold).

**Table 2** summarises co-authorship networks established by the grantees with regard to several network indicators. Junior and senior grantees differed in network indices pre- and post-grant award, with senior grantees having bigger, denser and more modular collaboration communities. In relative terms, both junior and senior grantees increased the size of the community (indicator “No. of different co-authors”) within which they were collaborating in the post-award period. There was no statistically significant difference in this indicator between the two groups, despite the fact that junior grantees had a greater increase in the number of publications (**Table 1**). This could be explained by the fact that junior grantees worked in more compact groups both in the pre- and post-award periods, as indicated by their higher network densities in comparison to senior grantees (**Table 2**). The amount of collaboration generated by publications was similar for both groups, as visible in the median number of co-authorships. There was a decrease in the network densities in the post-award period, significantly more pronounced for junior grantees. Post-award collaboration networks increased significantly more for junior grantees, as measured by the increase in network modularity, without a difference in the number of emerging communities between junior and senior grantees. All modularity values were beyond 0.3, the reference indicator of significant community structures in a network [32]. Senior grantees had higher modularity values (over 0.5) but junior grantees showed a greater increase.

**Table 2:** Co-authorship network indices (median, 95% confidence interval) for the publications in Scopus of junior (StG) and senior (AdG) ERC grantees in the pre- and post-award period^a^.

		StG (n = 184)			AdG (n = 171)			
Indicator	Pre-award	Post-award	Difference	Pre-award	Post-award	Difference	P^b^	BF_10_^c^
**No. of different co-authors**	39.0 (34.0, 45.0)	76.0 (63.0, 85.0)	33.0 (23.0, 40.0)	107.5 (89.0, 134.0)	161.5 (140.0, 199.0)	37.5 (27.0, 54.0)	0.150	2.14
**No. of co-authorships**	172.0 (139.0, 214.0)	422.0 (314.0, 544.0)	178.0 (110.0, 292.0)	659.5 (508.0, 842.0)	1370.0 (952.0, 1834.0)	403.5 (245.0, 718.0)	0.021	0.19
**Network density**	0.251 (0.230, 0.281)	0.176 (0.144, 0.194)	-0.076 (-0.092, -0.061)	0.118 (0.103, 0.131)	0.105 (0.089, 0.119)	-0.011 (-0.016, 0.0)	<0.001	**6.61x10**^**6**^
**No. of communities**	4.0 (3.0, 4.0)	5.0 (4.0, 5.0)	1.0 (1.0, 1.0)	6.0 (5.0, 6.0)	6.0 (6.0, 7.0)	0.0 (0.0, 1.0)	0.016	0.58
**Network modularity**	0.380 (0.355, 0.399)	0.473 (0.449, 0.491)	0.091 (0.066, 0.114)	0.517 (0.502, 0.531)	0.546 (0.515, 0.568)	0.026 (0.010, 0.402)	<0.001	**82.12**
**Grantee centrality**	0.388 (0.364, 0.408)	0.377 (0.350, 0.399)	-0.003 (-0.045, 0.017)	0.420 (0.369, 0.439)	0.338 (0.307, 0.370)	-0.036 (-0.057, -0.007)	0.041	0.380
**Network centralization**	0.671 (0.639, 0.698)	0.747 (0.733, 0.766)	0.083 (0.064, 0.103)	0.808 (0.794, 0.824)	0.827 (0.804, 0.835)	0.012 (-0.001, 0.023)	<0.001	**88x10**^**3**^

^a^Data from Scopus only. See Methods section and **Fig 1** for explanations about timeframes for data collection and explanation for co-authorship network indices.

^b^Comparison of median post- vs. pre-grant differences for StG and AdG, Mann-Whitney U test for independent samples.

^c^Comparison of median post- vs. pre-grant differences for StG and AdG, Bayesian t test for independent samples (significant differences in bold).

**Fig 2:**
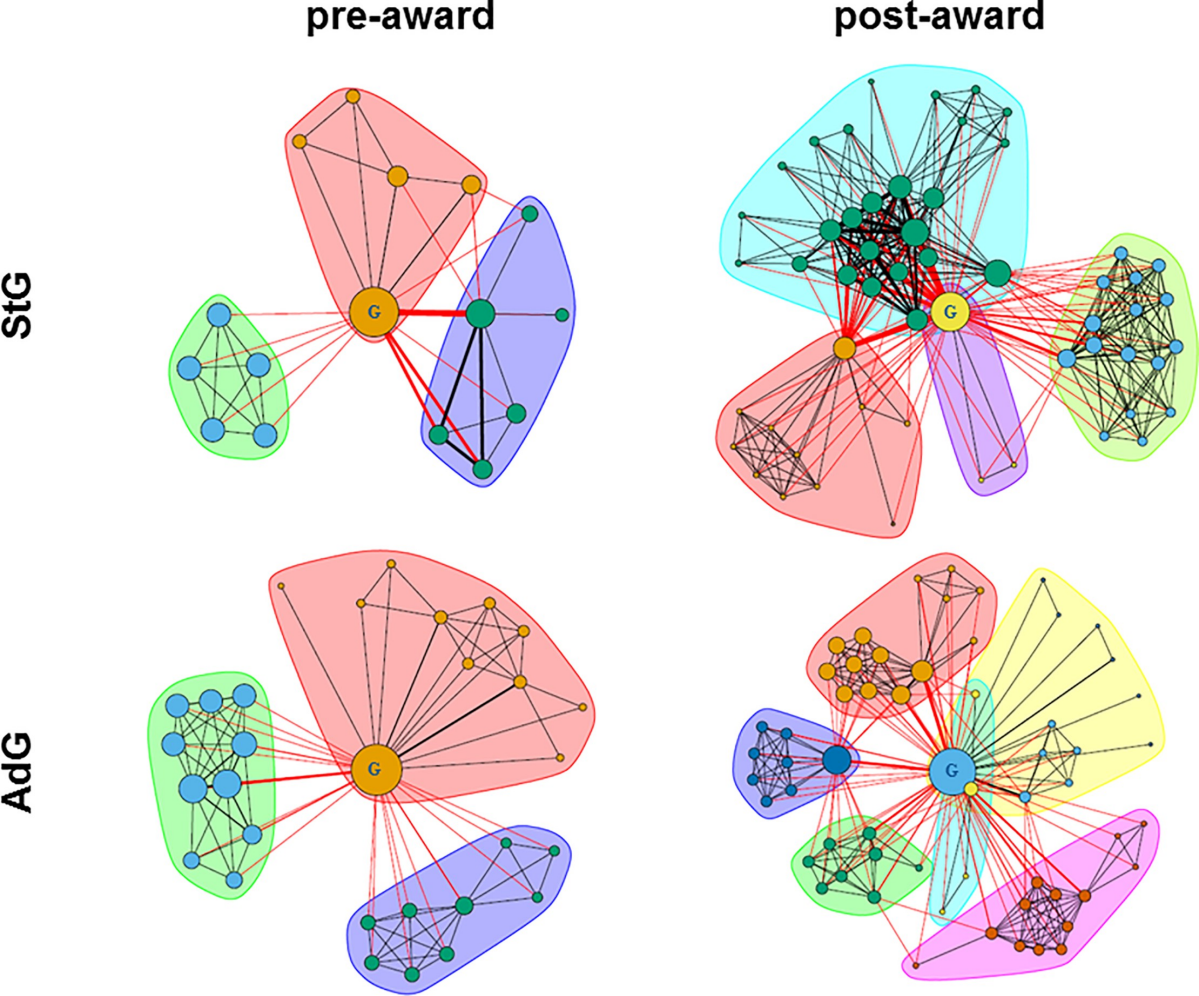
Collaboration (co-authorship) networks for two representative junior (StG) and senior (AdG) ERC grantees (placed in the centre of the graphs). Each researcher (co-author) is depicted as a node whose size refers to the eigencentrality score and, thus, to his or her relative importance within the network. The colour of the node is assigned automatically for each individual network and cannot be used for comparisons. Edge widths represent the number of publications co-authored by the two linked researchers. Edge colours refer to the inter-cluster or intra-cluster connectivity: black edges correspond to links within the same community whereas red edges connect co-authors that have been assigned to different communities. The presentations are based on the publication data from Scopus bibliographical and citation database.

**Fig 2** shows examples of pre- and post-award co-authorship networks for a junior and senior grantee to illustrate how the size of the collaboration network (number of nodes) and the number of sub-communities (coloured clusters) increased in the post-award period. In this example, the networks grew from 3 to 4 and from 3 to 6 communities for the junior and senior grantee, respectively. Also, the post-award co-authorship network represented a more structured collaboration pattern, where a variety of co-authors of different importance (node sizes) connect a more heterogeneous community (indicators “No. of communities” and “Network modularity” in **Table 2**). This effect was more pronounced for junior grantees, as they started from smaller and more compact pre-award co-authorship networks. The relative importance of the grantees within their community was reduced in the post-award period, mainly for senior grantees, who reduced their centrality in favour of other colleagues (**Table 2**). The post-award co-authorship networks shown in Fig 2 illustrate this situation, where higher centrality scores correspond to larger radius of nodes (emergence of researchers with intermediate importance). These nodes (researchers) became the link between the senior grantee and the others within the community, as reflected by the increase of post-award network modularity metrics. Due to the increase in the network heterogeneity (i.e., higher network modularity and lower grantee centrality), the overall network centralisation was augmented in a statistically meaningful way for junior grantees but not for seniors, who were already members of heterogeneous and robust collaboration networks in the pre-award period.

### Effect of gender

Both in pre- and post-award periods, male junior grantees had more publications, but no difference in citations per publication or percent manuscripts with last authorship (**Table 3**). This difference was not observed for senior male and female grantees either before or after the grant award. In terms of pre- and post-award differences, we could not identify gender differences except for a greater increase in the number of publications as last authors for female senior grantees. The differences between junior and senior grantees of the same gender followed the same pattern observed for the whole StG or AdG group shown in **Table 1**.

**Table 3:** Publication and citation output (median, 95% confidence interval) for female or male junior (StG) and senior (AdG) ERC grantees^a^.

	**Database**^b^	**Indicator**	**Male (N = 144)**			**Female (N = 40)**				
			**Pre-award**	**Post-award**	**Difference**	**Pre-award**	**Post-award**	**Difference**	**P**^c^	**BF**_**10**_^d^
**StG**	**Scopus**	**No. publications**	13.0 (11.0, 14.2)	21.0 (19.0, 23.2)	8.0 (6.0, 9.2)	9.0 (7.0, 11.0)	13.0 (10.0, 17.7)	5.0 (2.0, 8.7)	0.116	0.4
		**Citations per publication**	19.7 (17.0, 22.0)	16.1 (13.8, 19.0)	-1.9 (-5.1, -0.4)	18.3 (15.6, 22.0)	15.2 (10.0, 18.5)	-3.6 (-6.8, 1.4)	0.636	0.3
		**% last authorships**	20.0 (12.9, 25.0)	52.6 (50.0, 57.1)	21.6 (16.6, 29.9)	14.3 (8.9, 26.0)	47.2 (35.6, 63.8)	25.5 (14.0, 40.5)	0.646	0.2
	**WoS**	**No. publications**	12.0 (11.0, 13.2)	20.0 (18.0, 23.0)	7.0 (5.0, 9.0)	9.0 (7.0, 10.6)	13.0 (10.0, 16.7)	5.0 (2.0, 7.7)	0.189	0.3
		**Citations per publication**	18.2 (16.3, 21.6)	15.5 (12.9, 18.2)	-2.3 (-5.1, 0.0)	17.9 (14.9, 22.1)	14.1 (9.2, 17.9)	-4.1 (-6.6, 1.9)	0.405	0.2
		**% last authorships**	20.0 (14.1, 25.2)	52.0 (48.1, 55.1)	21.3 (16.1, 29.1)	17.4 (12.8, 26.5)	47.2 (37.5, 61.5)	31.6 (14.1, 44.3)	0.490	0.2
			**Male (N = 147)**			**Female (N = 24)**				
**AdG**	**Scopus**		**Pre-award**	**Post-award**	**Difference**	**Pre-award**	**Post-award**	**Difference**	**P**^b^	**BF**_**10**_^c^
		**No. publications**	33.0 (28.1, 39.8)	39.0 (34.1, 43.0)	4.0 (2.0, 7.0)	32.0 (26.0, 42.2)	32.0 (25.9, 43.2)	-3.0 (-6.5, 3.3)	0.006	2.5
		**Citations per publication**	20.4 (18.4, 22.4)	18.5 (16.9, 20.9)	-1.3 (-2.9, 0.1)	23.2 (19.5, 29.5)	19.1 (16.6, 27.4)	-1.1 (-7.2, 0.9)	0.489	0.4
		**% last authorships**	50.0 (46.7, 52.8)	46.3 (42.2, 50.0)	-0.1 (-0.1, 0.0)	52.5 (42.9, 59.8)	54.0 (47.3, 64.3)	3.6 (-1.8, 8.2)	0.006	**4.7**
	**WoS**	**No. publications**	32.0 (27.0, 38.0)	38.0 (32.0, 42.9)	3.0 (1.0, 6.0)	30.0 (24.8, 46.0)	30.5 (25.0, 41.2)	-2.0 (-5.5, 3.0)	0.013	2.1
		**Citations per publication**	19.8 (18.9, 21.7)	17.4 (15.7, 19.9)	-1.4 (-3.4, 0.4)	21.0 (18.8, 27.7)	17.6 (16.1, 25.9)	-3.3 (-7.3, 0.7)	0.353	0.5
		**% last authorships**	47.8 (46.0, 50.0)	46.7 (42.3, 50.0)	-4.9 (- 8.7, 3.3)	53.9 (42.6, 60.0)	52.9 (47.1, 62.7)	3.1 (-3.2, 10.9)	0.013	2.1

^a^See Methods section and **Fig 1** for explanations about timeframes for data collection.

^b^Scopus and Web of Science Core Collection (WoS).

^c^Comparison of median post- vs. pre-grant differences between male and female grantees, Mann-Whitney U test for independent samples.

^d^Comparison of median post- vs. pre-grant differences between male and female grantees, Bayesian t test for independent samples (significant differences in bold).

Gender differences were not observed in pre- or post-award indicators of scientific networking, except for greater number of different co-authors after grant award for male junior grantees (median of 80 vs 40 for female junior grantees) (**Table 4**). This gender difference was not observed for senior grantees. In terms of pre-post award difference, there were no significant gender differences in the indicators of scientific networking. Changes in network density and modularity, as well as in grantee centrality and network centralisation, were similar between female and male grantees and followed the general pattern observed for the whole StG or AdG group shown in **Table 2**.

**Table 4:** Co-authorship network indices (median, 95% confidence interval) for female or male junior (StG) and senior (AdG) ERC grantees^a^.

		**Males (N = 144)**			**Females (N = 40)**				
	**Indicator**	**Pre-award**	**Post-award**	**Difference**	**Pre-award**	**Post-award**	**Difference**	**P**^b^	**BF**_**10**_^c^
**StG**	**No. of different co-authors**	39.0 (35.0, 47.0)	80.0 (67.0, 99.0)	36.0 (26.0, 44.0)	33.5 (24.0, 39.0)	40.0 (34.0, 65.0)	14.0 (4.0, 39.0)	0.014	0.37
	**No. of co-authorships**	185.0 (145.0, 254.0)	474.0 (345.0, 632.0)	211.0 (145.0, 332.0)	134.0 (83.0, 214.0)	210.0 (137.0, 419.0)	81.5 (26.0, 218.0)	0.016	0.21
	**Network density**	0.241 (0.212, 0.268)	0.152 (0.136, 0.186)	-0.074 (-0.096, -0.054)	0.308 (0.248, 0.359)	0.221 (0.169, 0.245)	-0.084 (-0.129, -0.061)	0.352	0.28
	**No. of communities**	4.0 (3.0, 4.0)	5.0 (4.0, 5.0)	1.0 (1.0, 1.0)	4.0 (3.0, 4.0)	4.0 (4.0, 5.0)	1.0 (0.0, 2.0)	0.423	0.21
	**Network modularity**	0.390 (0.374, 0.421)	0.482 (0.456, 0.512)	0.097 (0.062, 0.119)	0.330 (0.277, 0.374)	0.400 (0.364, 0.473)	0.089 (0.047, 0.109)	0.594	0.27
	**Grantee centrality**	0.389 (0.362, 0.418)	0.377 (0.333, 0.401)	-0.015 (-0.054, 0.017)	0.385 (0.342, 0.433)	0.379 (0.341, 0.419)	0.012 (-0.034, 0.035)	0.498	0.31
	**Network centralization**	0.683 (0.643, 0.703)	0.763 (0.735, 0.783)	0.083 (0.062, 0.103)	0.642 (0.572, 0.671)	0.709 (0.673, 0.747)	0.084 (0.052, 0.122)	0.840	0.19
		**Males (N = 147)**			**Females (N = 24)**				
	**Indicator**^a^	**Pre-award**	**Post-award**	**Difference**	**Pre-award**	**Post-award**	**Difference**	**P**^b^	**BF**_**10**_^c^
**AdG**	**No. of different co-authors**	107.0 (91.0, 156.0)	165.5 (146.0, 249.0)	45.0 (34.0, 73.0)	118.5 (87.0, 179.0)	135.0 (98.0, 222.0)	11.0 (-11.0, 42.0)	0.005	0.31
	**No. of co-authorships**	651.5 (523.0, 1013.0)	1382.0 (1167.0, 2176.0)	475.0 (321.0, 927.0)	745.0 (475.0, 1898.0)	1023.0 (587.0, 2621.0)	97.5 (-194.0, 1040.0)	0.002	0.24
	**Network density**	0.117 (0.103, 0.137)	0.100 (0.092, 0.129)	-0.011 (-0.013, 0.03)	0.120 (0.096, 0.146)	0.107 (0.083, 0.164)	0.001 (-0.031, 0.024)	0.732	0.23
	**No. of communities**	6.0 (6.0, 7.0)	6.0 (6.0, 7.0)	0.0 (0.0, 2.0)	6.0 (5.0, 7.0)	6.0 (4.0, 9.0)	1.0 (-1.0, 3.0)	0.927	0.24
	**Network modularity**	0.516 (0.507, 0.542)	0.546 (0.529, 0.575)	0.027 (0.021, 0.048)	0.523 (0.471, 0.555)	0.542 (0.418, 0.606)	0.032 (-0.054, 0.097)	0.859	0.28
	**Grantee centrality**	0.427 (0.407, 0.450)	0.335 (0.314, 0.380)	-0.042 (-0.057, 0.018)	0.357 (0.288, 0.432)	0.399 (0.225, 0.421)	0.002 (-0.006, 0.068)	0.224	0.25
	**Network centralization**	0.808 (0.795, 0.838)	0.828 (0.818, 0.844)	0.015 (0.005, 0.025)	0.806 (0.777, 0.851)	0.815 (0.726, 0.849)	0.003 (-0.021, 0.053)	0.902	0.38

^a^Data from Scopus only. See Methods section and **Fig 1** for explanations about timeframes for data collection and explanation for co-authorship network indices.

^b^Comparison of median post- vs. pre-grant differences between male and female grantees, Mann-Whitney U test for independent samples.

^c^Comparison of median post- vs. pre-grant differences between male and female grantees, Bayesian t test for independent samples (significant differences in bold).

### Effect of geography

Junior grantees from higher and lower research performing countries did not differ in publication indicators either before or after the grant award (**Table 5**). For senior grantees, those from higher research performing countries had more publications with last authorships than senior grantees from lower research performing countries. The changes between the pre- and post-award periods in terms of publications were similar for the senior grantees, irrespective of the country group. On the other hand, junior grantees from higher research performing countries had a greater increase in their number of publications compared with those from lower research performing countries.

**Table 5:** Publication and citation output (median, 95% confidence interval) for junior (StG) and senior (AdG) ERC grantees from countries with higher or lower research performance^a^.

	**Database**^b^	**Indicator**	**Higher performance countries (N = 149)**			**Lower performance countries (N = 35)**				
			**Pre-award**	**Post-award**	**Difference**	**Pre-award**	**Post-award**	**Difference**	**P**^c^	**BF**_**10**_^d^
**StG**	**Scopus**	**No. publications**	11.0 (10.0, 13.0)	20.0 (18.1, 23.0)	8.0 (7.0, 9.0)^d^	13.0 (10.2, 15.0)	16.0 (11.2, 22.5)	5.0 (- 1.8, 6.0)	<0.001	**46.8**
		**Citations per publication**	20.1 (17.6, 22.0)	15.8 (13.9, 18.4)	-3.1 (-5.1, -1.0)	16.2 (12.2, 24.3)	14.0 (10.6, 22.7)	- 1.3 (-6.9, 1.9)	0.307	0.6
		**% last authorships**	18.2 (12.6, 25.0)	53.8 (50.0, 57.8)	23.8 (16.7, 32.2)	18.8 (7.3, 26.3)	47.4 (40.4, 55.3)	20.5 (10.9, 35.5)	0.762	0.2
	**WoS**	**No. publications**	11.0 (10.0, 12.0)	20.0 (17.0, 22.0)	7.0 (6.0, 9.0)^d^	13.0 (9.2, 16.0)	16.0 (12.4, 22.8)	4.0 (-1.8, 6.0)	0.004	**13.6**
		**Citations per publication**	18.5 (16.8, 21.5)	15.4 (13.7, 17.9)	-3.2 (-4.9, 1.7)	14.7 (11.4, 22.6)	11.5 (8.5, 21.4)	-2.1 (-7.3, 1.0)	0.541	0.4
		**% last authorships**	18.2 (14.3, 25.0)	52.2 (48.2, 57.1)	25.5 (17.3, 31.5)	20.0 (8.3, 28.0)	46.2 (33.3, 54.8)	16.7 (6.7, 35.1)	0.477	0.2
			**Higher performance countries (N = 150)**			**Lower performance countries (N = 21)**				
**AdG**	**Scopus**		**Pre-award**	**Post-award**	**Difference**	**Pre-award**	**Post-award**	**Difference**	**P**^b^	**BF**_**10**_^c^
		**No. publications**	32.0 (28.5, 37.0)	36.0 (31.6, 40.4)	3.0 (0.0, 5.0)	44.0 (22.7, 60.2)	44.0 (35.6, 58.8)	4.0 (-2.4, 9.6)	0.747	0.2
		**Citations per publication**	20.4 (18.7, 22.2)	18.9 (17.1, 20.6)	- 1.2 (-2.7, 0.1)	23.2 (19.7, 25.8)	17.8 (13.4, 33.6)	-2.7 (-9.6, 8.1)	0.851	0.4
		**% last authorships**	51.7 (48.0, 56.0)	50.0 (45.3, 52.5)	-4.5 (-7.3, 0.0)	40.4 (32.9, 46.6)	37.7 (30.6, 50.0)	2.8 (-7.9, 11.0)	0.202	0.5
	**WoS**	**No. publications**	31.0 (27.0, 35.4)	33.5 (30.0, 40.0)	2.0 (0.0, 5.0)	41.0 (22.1, 57.5)	43.0 (34.8, 56.4)	3.0 (-2.0, 15.2)	0.556	0.3
		**Citations per publication**	19.7 (19.0, 21.1)	17.5 (16.5, 19.2)	-1.6 (-3.3, 0.4)	23.2 (16.2, 26.8)	16.9 (12.9, 30.9)	-4.2 (-9.2, 6.2)	0.944	0.3
		**% last authorships**	50.0 (47.0,53.0)	50.0 (45.1,52.1)	-4.6 (-7.6,-2.1)	42.9 (33.3, 46.6)	37.1 (30.6,51.1)	0.0 (-10.3, 9.0)	0.404	0.4

^a^See Methods section and **Fig 1** for explanations about timeframes for data collection.

^b^Scopus and Web of Science Core Collection (WoS).

^c^Comparison of median post- vs. pre-grant differences between grantees from higher and lower research performing countries, Mann-Whitney U test for independent samples.

^d^Comparison of median post- vs. pre-grant differences between grantees from higher and lower research performing countries, Bayesian t test for independent samples (significant differences in bold).

**Fig 3:**
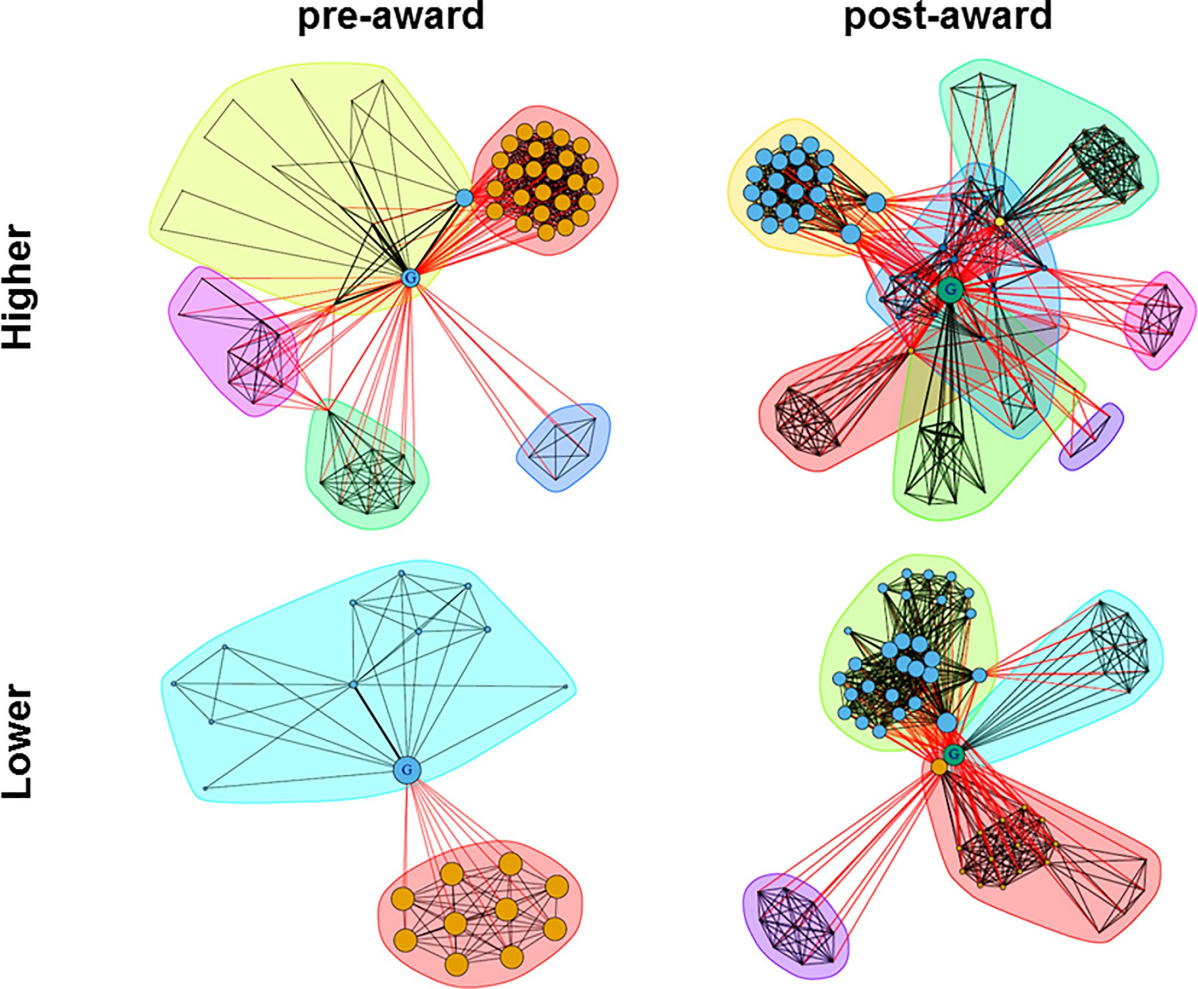
**Collaboration (co-authorship) networks for two representative junior (StG) ERC grantees (placed in the centre of the graphs) from higher (top) and lower (bottom) performing countries.** Each researcher (co-author) is depicted as a node whose size refers to the eigencentrality score and, thus, to his or her relative importance within the network. The colour of the node is assigned automatically for each individual network and cannot be used for comparisons. Edge widths represent the number of publications co-authored by the two linked researchers. Edge colours refer to the inter-cluster or intra-cluster connectivity: black edges correspond to links within the same community whereas red edges connect co-authors that have been assigned to different communities. The presentations are based on the publication data from Scopus bibliographical and citation database.

The location of the researcher’s host organisation had an influence on the evolution of co-authorship networks only for junior grantees (**Table 6**). Junior grantees from higher research-performing countries increased the number of different co-authors after the grant award in comparison to their colleagues from lower research-performing countries; this difference was significant on frequentist but not Bayes statistical analysis. Furthermore, while all junior grantees expanded their collaboration networks (more co-authors and co-authorships), those located in higher research-performing countries became a part of more diverse (lower density) and robust (higher centralisation) networks than their counterparts from lower research-performing countries. This is illustrated with the examples of two junior grantees in **Fig 3**. Senior grantees from higher and lower research performing countries did not differ in pre- and post-award network indices and experienced similar pre-post award changes in these indices (**Table 6**).

**Table 6:** Co-authorship network indices (median, 95% confidence interval) for junior (StG) and senior (AdG) ERC grantees from countries with higher or lower research performance^a^.

		**Higher performance countries (N = 149)**			**Lower performance countries (N = 35)**				
	**Indicator**	**Pre-award**	**Post-award**	**Difference**	**Pre-award**	**Post-award**	**Difference**	**P**^b^	**BF**_**10**_^c^
**StG**	**No. of different co-authors**	38.5 (34.0, 43.0)	80.0 (63.0, 100.0)	37.5 (26.0, 49.0)	39.0 (29.0, 63.0)	66.0 (55.0, 78.0)	15.0 (4.0, 33.0)	0.003	0.57
	**No. of co-authorships**	160.0 (139.0, 241.0)	443.0 (308.0, 631.0)	210.5 (114.0, 317.0)	196.0 (123.0, 268.0)	353.0 (266.0, 470.0)	139.0 (33.0, 238.0)	0.078	0.22
	**Network density**	0.252 (0.233, 0.290)	0.166 (0.141, 0.189)	-0.079 (-0.102, -0.067)	0.238 (0.187, 0.282)	0.210 (0.140, 0.229)	-0.014 (-0.074, 0.008)	<0.001	**4.95**
	**No. of communities**	4.0 (3.0, 4.0)	5.0 (4.0, 5.0)	1.0 (1.0, 2.0)	4.0 (3.0, 5.0)	4.0 (4.0, 5.0)	1.0 (0.0, 1.0)	0.036	1.22
	**Network modularity**	0.378 (0.348, 0.392)	0.481 (0.453, 0.502)	0.114 (0.085, 0.130)	0.390 (0.350, 0.454)	0.455 (0.353, 0.478)	0.039 (-0.009, 0.052)	<0.001	2.36
	**Grantee centrality**	0.382 (0.357, 0.404)	0.384 (0.341, 0.402)	0.008 (-0.032, 0.032)	0.420 (0.366, 0.450)	0.365 (0.332, 0.418)	-0.053 (-0.084, 0.007)	0.109	0.28
	**Network centralization**	0.664 (0.614, 0.689)	0.754 (0.735, 0.780)	0.092 (0.074, 0.121)	0.708 (0.651, 0.736)	0.716 (0.677, 0.782)	0.010 (-0.010, 0.054)	<0.001	**5.34**
		**Higher performance countries (N = 150)**			**Lower performance countries (N = 21)**				
	**Indicator**	**Pre-award**	**Post-award**	**Difference**	**Pre-award**	**Post-award**	**Difference**	**P**^b^	**BF**_**10**_^c^
**AdG**	**No. of different co-authors**	103.0 (85.0, 120.0)	157.0 (126.0, 193.0)	37.0 (27.0, 54.0)	194.0 (75.0, 276.0)	261.5 (101.0, 412.0)	40.5 (1.0, 133.0)	0.917	0.25
	**No. of** **co-authorships**	627.0 (481.0, 822.0)	1296.0 (911.0, 1618.0)	403.5 (230.0, 739.0)	1318.0 (395.0, 2339.0)	2228.0 (804.0, 3999.0)	407.5 (-12.0, 1792.0)	0.967	0.67
	**Network** **density**	0.119 (0.103, 0.131)	0.107 (0.093, 0.121)	-0.008 (-0.015, 0.03)	0.110 (0.061, 0.168)	0.079 (0.048, 0.151)	-0.015 (-0.027, 0.008)	0.167	0.35
	**No. of communities**	6.0 (5.0, 6.0)	6.0 (6.0, 7.0)	0.0 (0.0, 1.0)	5.5 (4.0, 9.0)	8.0 (7.0, 11.0)	1.5 (0.0, 3.0)	0.022	1.48
	**Network modularity**	0.519 (0.501, 0.533)	0.535 0.511, 0.563)	0.024 (0.007, 0.037)	0.510 (0.319, 0.540)	0.577 (0.428, 0.667)	0.057 (-0.003, 0.099)	0.101	1.21
	**Grantee centrality**	0.422 (0.369, 0.439)	0.335 (0.305, 0.369)	-0.042 (-0.072, -0.007)	0.410 (0.220, 0.476)	0.367 (0.270, 0.415)	-0.016 (-0.080, 0.019)	0.330	0.47
	**Network centralization**	0.808 (0.795, 0.824)	0.822 (0.796, 0.834)	0.007 (-0.006, 0.023)	0.808 (0.675, 0.854)	0.847 (0.755, 0.898)	0.023 (-0.001, 0.037)	0.167	0.49

^a^Data from Scopus only. See Methods section and **Fig 1** for explanations about timeframes for data collection and explanation for co-authorship networks.

^b^Comparison of median post- vs. pre-grant differences between grantees from higher and lower research performing countries, Mann-Whitney U test for independent samples.

^c^Comparison of median post- vs. pre-grant differences between grantees from higher and lower research performing countries, Bayesian t test for independent samples (significant differences in bold).

## Discussion

Our study demonstrated that seniority, gender and place of work are associated with the publication output and collaboration networks of ERC grantees, particularly for junior ones. Before the grant award, male junior grantees had more publications than female junior grantees and junior grantees from higher research performing countries collaborated with more other researchers that those from lower-research performing countries. In relation to the change in their performance from before the grant award to five years after the grant award, there were no major gender differences among both junior ERC grantees, although those working in lower research-performing countries did not publish and develop their collaboration networks to the same extent as their peers in higher research-performing countries. Gender and country differences were not observed for senior grantees to the same extent as for junior grantees. Senior grantees had significantly greater publication output than the junior grantees, both before and after the grant award. However, junior grantees had a greater increase in publications and last (senior) authorships. The post-award collaboration networks size and the number of sub-communities increased for both junior and senior grantees, but this change was greater for junior grantees, as they departed from smaller and more compact pre-award co-authorship networks.

The results of our study should be interpreted with potential limitations in mind. As we did not have access to the (non-public) data on unsuccessful grantees, we were not able to compare the grantees with a control group of researchers with similar profiles but lacking ERC funding. Similarly, it would also be interesting to check if different types of funding sources affect similarly the bibliometric outputs and collaboration networks or if it favours any cumulative advantage [37]. The groups compared by gender and geographical location were not comparable in size because there were much fewer women and researchers from lower research-performing countries as recipients of ERC grants. Because of the groups’ inequality, we used Bayes statistics due to its advantage of coherency and independency of the intention with which data are collected, as well as a minimum bias towards the null hypothesis [35]. We also did not explore the possible influence of the research field on the publication performance and collaboration networks. The subgroup analysis of the 9 panels from the ERC life sciences domain was not meaningful because of small number of grantees in some panels. However, recent studies of ERC panel review decision-making processes indicate that there are gender differences, which may be related to the social dynamics in different panels [38, 39].

An important finding of our study was the value of collaboration network analysis in assessing grant publication performance change, particularly in comparison to standard bibliometric indicators of publications and citations. This seems to be particularly true for researchers who were already highly productive before the grant award, such as those competing for advanced ERC grants.

The differences we observed between junior and senior grantees and the differences between the pre- and post-award periods for both grantee groups corroborate the value of combining bibliometric and collaboration networks analysis to assess scientific collaborative trends. Junior researchers benefited greatly from their ERC grants, confirmed by both the increase of number of publications, especially last author publications, and the expansion of their collaboration networks. They also positioned themselves within their respective scientific communities. For senior researchers, we observed that the centrality of their collaborative networks decreased after the grant. Such decentralisation can be understood as a positive effect for a growing scientific community because it results in more robust collaboration networks. This effect is also supported by the observation of an overall decrease in the proportion of publications as last author for senior grantees. A decentralised community can stay connected despite the disappearance of some of the nodes, which increases the robustness of the networks. This is similar to the way the Internet remains stable because there is no central server, but rather a decentralised structure, with many nodes on multiple receiving and forwarding networks [40].

While we observed gender differences both before and after the grant award in the number of publications, with male grantees publishing more papers than female grantees, the pre-post change in the publication output was similar for the two genders, both for junior and senior grantees. Senior female researchers, in contrast to their male counterparts, even increased their proportion of publications as last authors. The lack of major differences between male and female grantees in the post-award change in their publishing productivity and collaborative patterns suggests that researchers awarded an ERC grant, irrespective of their gender, have comparable output from the moment of grant award. Still, ERC granting results in significant underrepresentation of women (only 18% of our sample), but this may be related more to lower propensity in submitting grants proposals than to their research performance [41, 42], and biased peer review process [38, 39].

The place of work of the researchers also seemed to influence grant performance, both in terms of publications and positioning in the global scientific community. Junior grantees in lower research-performing countries performed worse than their peers in higher research-performing countries, suggesting that the environment in lower research-performing countries may not be beneficial for their career development, possibly due to the lack of additional support or leverage effects disposed at the regional or national level. On the other hand, senior grantees from lower research performing countries already had a higher publication track record before the award of the grant. It is important to keep in mind that the number of grantees from lower research performing countries was more than 3 times smaller than those from high research performing countries, reflecting also the difference in the applications for ERC grants: researchers from higher research performing countries represent the vast majority of applicants for ERC grants [43].

Our study is a preliminary study of publicly available data on ERC grants. It would be important to test the usefulness of collaboration networks analysis on full proposal datasets that include not only the grantees but also the (non-successful) applicants, as done in other granting systems. Funding agencies should follow the example of journals [44] and open their grant peer review data to meta-research in order to learn more about their processes, to develop better measures to validate peer review, and to evaluate grant success. Network analysis may add valuable information to standard publication and citation outputs, by providing insights not only into the structure of knowledge but also into the structure of the research community [24], which is linked to research performance [10, 45].
